# Differences in Whole-Blood Transcriptional Profiles in Inflammatory Bowel Disease Patients Responding to Vedolizumab Compared with Non-Responders †

**DOI:** 10.3390/ijms24065820

**Published:** 2023-03-18

**Authors:** Sofie Haglund, Jan Söderman, Sven Almer

**Affiliations:** 1 Department of Biomedical and Clinical Sciences, Linköping University, 581 83 Linköping, Sweden; jan.soderman@rjl.se; 2 Laboratory Medicine, Region Jönköping County, 551 85 Jönköping, Sweden; 3 IBD-Unit, Division of Gastroenterology, Karolinska University Hospital, 171 76 Stockholm, Sweden; sven.almer@ki.se; 4 Department of Medicine, Karolinska Institutet—Solna, 171 76 Stockholm, Sweden

**Keywords:** vedolizumab, biomarker, differentially expressed genes, gene set enrichment analysis, RNA-seq, RNA sequencing, transcriptome

## Abstract

Vedolizumab is efficacious in the treatment of Crohn’s disease (CD) and ulcerative colitis (UC). However, a significant proportion of patients present with a non-response. To investigate whether differences in the clinical response to vedolizumab is reflected in changes in gene expression levels in whole blood, samples were collected at baseline before treatment, and at follow-up after 10–12 weeks. Whole genome transcriptional profiles were established by RNA sequencing. Before treatment, no differentially expressed genes were noted between responders (*n* = 9, UC 4, CD 5) and non-responders (*n* = 11, UC 3, CD 8). At follow-up, compared with baseline, responders displayed 201 differentially expressed genes, and 51 upregulated (e.g., translation initiation, mitochondrial translation, and peroxisomal membrane protein import) and 221 downregulated (e.g., Toll-like receptor activating cascades, and phagocytosis related) pathways. Twenty-two of the upregulated pathways in responders were instead downregulated in non-responders. The results correspond with a dampening of inflammatory activity in responders. Although considered a gut-specific drug, our study shows a considerable gene regulation in the blood of patients responding to vedolizumab. It also suggests that whole blood is not optimal for identifying predictive pre-treatment biomarkers based on individual genes. However, treatment outcomes may depend on several interacting genes, and our results indicate a possible potential of pathway analysis in predicting response to treatment, which merits further investigation.

## 1. Introduction

The need for personalized medicine has become increasingly more evident in the treatment of inflammatory bowel disease (IBD) in view of the complex immunoregulation and an expanding repertoire of drugs available. The use of effective drugs, such as immunomodulatory thiopurines and biologics, is associated with a significant risk of toxicity and with considerable costs. Therefore, biomarkers are strongly needed to guide medical decisions concerning when to use these drugs. The most efficient biological treatment used in IBD is traditionally considered to be treatment with TNF-α antagonists (adalimumab, certolizumab, golimumab, infliximab) in combination with thiopurines [1,2]. More recently, biological drugs targeting other molecules involved in the immune system, such as antibodies inhibiting the p40 subunits of IL-12 and -23 (ustekinumab) and α_4_β_7_ integrins on leukocytes (vedolizumab, VDZ), have been developed and have entered into clinical routine use [2,3,4].

The trafficking of leukocytes into the inflamed intestinal tissue is a driver of inflammation and a highly regulated process. T cells present in circulating blood enter the intestinal mucosa through the interaction between the α_4_β_7_ integrin expressed on their surface and the mucosal vascular addressin cell adhesion molecule (MAdCAM-1) on the vascular endothelium [5].

VDZ is a humanized antibody which is generally considered to be gut-specific and to specifically target this interaction, thereby reducing the T cell infiltration in the intestinal tissue. VDZ may be an alternative in patients who have failed to response anti-TNF-α treatment and who often present progressive forms of disease. However, despite efficacy in a large proportion of patients, subgroups show persistent disease activity [6,7,8,9]. Thus, stratified approaches are needed in order to deliver an appropriate treatment.

Transcriptional profiling of intestinal biopsies has shown that endoscopic healing following VDZ treatment is associated with the regulation of immune-related genes involved in leukocyte migration and lipid metabolism in the intestinal mucosa [10]. The effect has been found to be most pronounced after one year of VDZ therapy. Clinical remission following VDZ treatment has been associated with a dampening of the innate immune response, granulocyte adhesion and diapedesis, as well as with TNF signalling pathways [11,12]. The trough concentration of VDZ, the expression and saturation of α_4_β_7_-integrin on peripheral blood CD4+ T cells, as well as on mucosal CD4+ cells, have been suggested as promising predictive biomarkers for treatment guidance, even though controversy exists [12,13,14,15]. Furthermore, the serum concentration of chemokines has been investigated in relation to the clinical response to treatment [16,17]. However, the results of these studies have not made their way into clinical practice.

Even though VDZ is considered gut-specific, its effects in vivo have not been fully elucidated, which calls for investigations. Here, we hypothesized that differences in clinical response to VDZ therapy might be reflected in changes in gene expression levels in whole blood, which, in contrast to intestinal biopsies and isolated cell populations, could provide easily accessible biomarkers. RNA-sequencing data were investigated for differentially expressed genes (DEGs) and for gene set enrichment in biological pathways. We show a large number of DEGs following VDZ in responders and suggest that pathway analysis may provide a potential tool for predicting responses to treatment, although further studies are needed.

## 2. Results

### 2.1. Clinical Characteristics

Overall, routine blood laboratory parameters were unchanged following treatment with VDZ (T1) when compared with baseline (T0), both in responders (*n* = 13) and in non-responders (*n* = 11). However, the serum concentration of C-reactive protein (CRP) was higher in responders compared with non-responders at both time points. The concentration did not change over time in any group (Table 1). Four responders and four non-responders were able to withdraw corticosteroids following the introduction of VDZ.

The clinical disease activity indices; the simplified Harvey Bradshaw index (sHBI) and the Simple Clinical Colitis Activity Index (SCCAI) correlated with the ‘physician global assessment’ (PGA): sHBI r_s_ = 0.65, *p* = 0.0002, and SSCAI r_s_ = 0.84, *p* < 0.0001, when data from both T0 and T1 were included. 

The proportion of patients treated with anti-TNF-α drugs during the six months preceding VDZ were similar in responders (58%) and non-responders (64%), (*p* = 1.00), as was the time elapsed between withdrawal of the last anti-TNF-α drug and the introduction of VDZ (*p* = 0.98) (Table 1).

At the 6 month follow-up after the initial three infusions, 19/24 (79%) of patients were still on VDZ treatment: 11/13 (85%) among responders and 8/11 (73%) among non-responders (*p* = 0.63). At 12 months, 15/24 (63%) of patients were still on VDZ treatment: 10/13 (77%) among responders and 5/11 (45%) among non-responders, *p* = 0.21. The main reason for withdrawal was persisting disease activity.

### 2.2. Plasma Concentration of VDZ vs. Response to Treatment

There was no difference in the plasma concentrations of VDZ between responders and non-responders at T1 (Table 1). The concentration of VDZ in CD patients [14.2 (21.8) µg/mL] was comparable to that in UC patients [9.6 (14.1) µg/mL] (*p* = 0.07).

### 2.3. Whole-Genome Expression Analysis

RNA, both at baseline (T0) and at follow-up (T1), was available for 20 patients, 9 responders and 11 non-responders (Table S1). The number of reads per sample was 25.3 (range 10.4–65.6) million. Out of a total of 56,303 genes, 12,666 remained for statistical analysis after filtering. 

Multidimensional scaling suggested group differences based on gender (separation along dimension 1, *p* = 5.7 × 10^−3^; separation along dimension 2, *p* = 3.6 × 10^−10^). When comparing females (*n* = 6) with males (*n* = 14) at baseline (T0), 33 DEGs were observed, and at the follow-up (T1) 30 DEGs were seen. There was an overlap between the genes identified at T0 and T1, represented by 28 genes located at the X- or Y-chromosome, and by removing sex chromosome-related genes prior to multidimensionally scaling the observed clustering of samples eliminated. In addition, the gender distributions between the major study groups (responders and non-responders) were similar. No grouping of samples based on disease type, whether UC or CD, was observed (separation along dimension 1, *p* = 0.57, separation along dimension 2, *p* = 0.33). Therefore, further analyses were performed without stratification based on disease type or gender.

### 2.4. Deconvolution 

When RNA sequencing data were used for the estimation of the relative abundance of white blood cells, a decrease in the abundance of B cells was observed in responders at follow-up (T1) compared with baseline (T0), with *p* = 0.046 (Table S2).

### 2.5. Gene-Expression in Responders Compared with Non-Responders

No DEGs were observed between responders (*n* = 9) and non-responders (*n* = 11), neither when analysing transcriptional profiles at baseline (T0), nor were any detected at follow-up (T1) under treatment (Table S3: Sheets 1–2).

### 2.6. VDZ Regulates the Expression of Genes in Whole Blood Only in Patients Responding to Treatment

**Figure 1:**
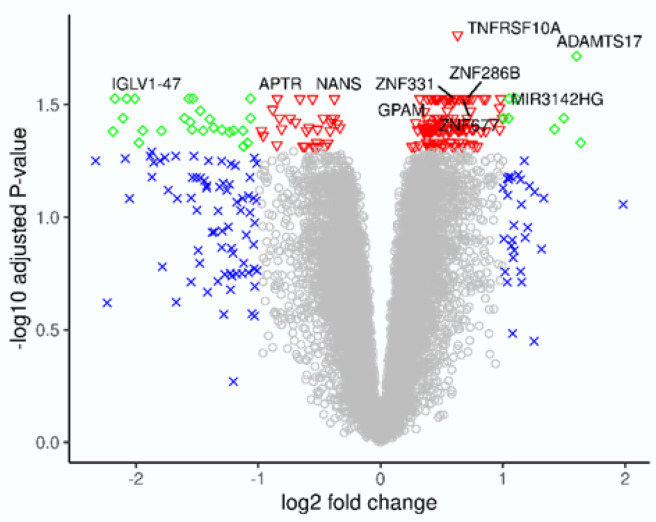
In responders (*n* = 9), 201 genes were regulated following treatment with vedolizumab compared with baseline. The 10 most significant differentially expressed genes are annotated. ◊ indicates FDR < 0.05 and absolute value of FC > 2; ∇ FDR < 0.05 and absolute value of FC < 2; x FDR > 0.05 and absolute value of FC > 2, ° FDR > 0.05 and FC < 2; FDR: false discovery rate-adjusted *p* value; FC: fold change.

When transcriptional profiles at follow-up (T1), were compared with those at baseline (T0) in responders (*n* = 9), 201 genes were regulated, of which 13 were pseudogenes (Figure 1, Table S3: Sheet 3). Twenty-five of the 55 downregulated genes were regulated at least two-fold. The majority of them were immunoglobulin light-chain and immunoglobulin heavy-chain genes (e.g., *IGHV3-11, IGHA2, IGLV1-47,* and *IGLV1-44*). The gene *MZB1* is ascribed many functions. It helps to diversify B-cell functions, promotes IgM assembly and secretion, and may also function as a proinflammatory cytokine. An isoform of *MZB1* may be involved in the regulation of apoptosis.

Eight of the 146 upregulated genes were regulated at least two-fold. These genes mainly represented the processes involved in the suppression of the non-canonical pathways of NFKB activation (*OLFM1*), in T-cell receptor alpha joining (*TRAJ7*, *TRAJ11, TRAJ18*)*,* in processes involving disintegrin and metalloproteinases with thrombospondin motifs such as the modulation of the bone morphogenetic protein, the SMAD1/5/8 signaling pathway (*ADAMTS17*), in proto-oncogenic functions (*HRAS*), in voltage-dependent Ca^2+^ channel signaling (*CACNG8*), and in the regulation of the glucose metabolism and lipogenesis (*XYLB*), as described by UniProtKB. 

**Figure 2:**
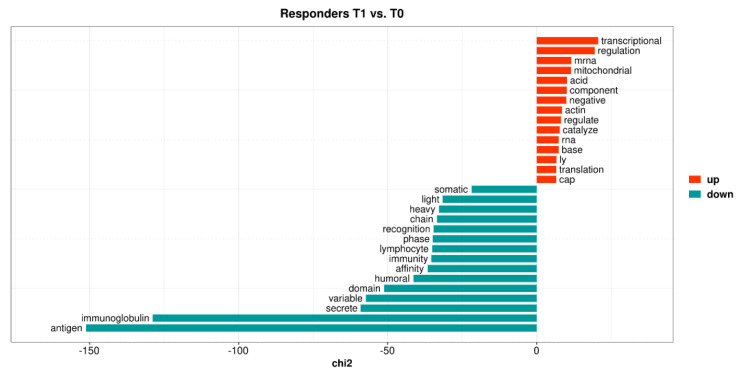
The top 15 keyness words describing a preferential association of unigrams, with either upregulated (red bars) or downregulated (blue-green bars) genes in responders when comparing follow-up samples of vedolizumab treatment with baseline. Keyness words were based on the textual analysis of functional protein information (UniProtKB) associated with the differentially expressed genes. The chi2 value on the *x*-axis is the test statistic obtained from Chi-square test used to calculate keyness.

An over-representation analysis identified no over-represented biological pathways among the 201 DEGs (Table S3, Sheet 4). However, using quantitative text analysis (169 out of 188 non-pseudogenes were represented by proteins in UniProtKB), it was determined that the term mitochondria was over-represented among the upregulated genes, whereas immunoglobulin was found to be over-represented among the downregulated genes (Figure 2). 

No DEGs were observed in non-responders (*n* = 11) when follow-up data under VDZ treatment (T1) were compared with those at baseline (T0) (Table S3: Sheet 5).

### 2.7. Gene Set Enrichment Analyses 

#### 2.7.1. Enrichment Analysis in Responders Compared with Non-Responders

Next we used the entire dataset for gene set enrichment analysis in order to detect subtle and coordinated alterations in gene expression. 

**Figure 3:**
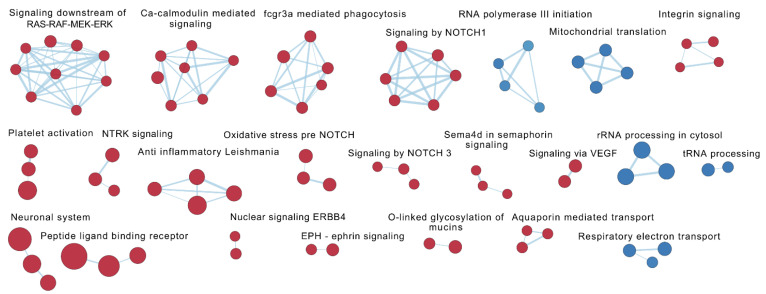
Visualization of common contexts of the enriched reactome pathways based on the statistical comparison of transcriptional profiles between responders (*n* = 9) and non-responders (*n* = 11) at baseline (T0). The enrichment map shows pathways with a normalized absolute value of the enrichment score ≥ 2 and/or interacting with other pathways. Red; pathways upregulated in responders, Blue; pathways downregulated responders. Darker colour represents a more positive (red) or negative (blue) score.

At baseline (T0), 325 reactome pathways were differentially regulated (FDR < 0.075) in responders (*n* = 9) compared with non-responders (*n* = 11) (Table 2). The most upregulated pathways in responders concerned themes of “innate immunity”, “phagocytic processes”, “cytoskeleton modulation”, “amino acid transport across the plasma membrane”, and “glycosaminoglucan metabolism”. Among the most downregulated pathways were “mitochondrial processes” such as “mitochondrial translation” and “respiratory electron transport”, “processing of rRNA” and “processing of tRNA” (Figure 3, Table 3 and Table S4; Sheet 1).

**Figure 4:**
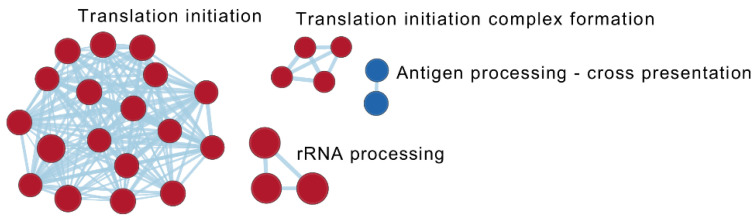
Visualization of common contexts of the enriched reactome pathways based on the statistical comparison of transcriptional profiles between responders (*n* = 9) and non-responders (*n* = 11) at the time of follow-up (T1). Pathways with a normalized absolute value of the enrichment score ≥ 2 and/or interacting with another pathway are shown. Red: pathways upregulated in responders; Blue: pathways downregulated in responders. Darker colour represents a more positive (red) or negative (blue) score.

At follow-up, the difference between responders and non-responders was represented by 40 reactome pathways (Table 2). The most significant upregulated pathways in responders were related to “translation” and its regulation, while pathways associated with the themes “antigen processing-cross presentation” (antigen cross-presentation/cross-priming of exogenous antigens on MHC class I molecules to CD8+ T lymphocytes), “ADP signaling through P2Y purinoceptor 12” as well as “interferon α, β, and γ signaling” were the most downregulated (Figure 4, Table 4 and Table S4; Sheet 2).

The pathways for “rRNA processing”, “rRNA modification in nucleolus and cytosol”, “major pathway of rRNA processing in nucleus and cytosol”, and “rRNA processing” were downregulated at baseline (T0) in responders (negative normalized enrichment score) compared with non-responders. However, the same pathways were upregulated at follow-up (positive normalized enrichment score).

#### 2.7.2. The Effect of VDZ Treatment on Biological Pathways within Responders and Non-Responders

**Figure 5:**
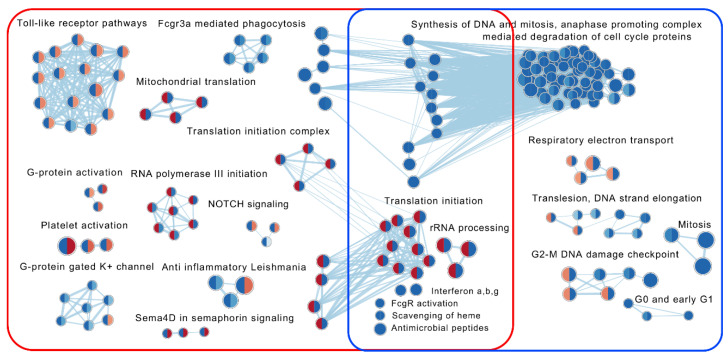
Illustration of common contexts of enriched reactome pathways based on the statistical comparisons between transcriptional profiles at the time of follow-up with VDZ (T1) and baseline (T0) in responders (*n* = 9) and non-responders (*n* = 11), respectively. The enrichment map shows pathways with an absolute normalized enrichment score ≥ 2 and/or interacting with more than two other pathways. Darker colour represents a more positive (red) or negative (blue) score. Each node is divided into two halves, in which the left half represents T1 vs. T0 in responders and the right half represents T1 vs. T0 in non-responders. Nodes associated with the transcriptional dynamics in responders and non-responders are encircled in red and blue, respectively (for further details see Table S4; Sheets 3–5).

Under treatment with VDZ, 272 and 194 reactome pathways were differentially regulated (FDR < 0.075) compared with baseline in responders and in non-responders, respectively (Figure 5, Table 2 and Table S4; Sheets 3–5). Of the 51 reactome pathways uniquely upregulated in responders, 22 were instead downregulated in non-responders (e.g., pathways of “rRNA processing in the nucleolus and cytosol”, “translation initiation”, “translation elongation”, “translation termination”, “mitochondrial translation elongation and termination”, and “peroxisomal membrane protein import”). Fourteen of these pathways were among the top 25 most significantly upregulated pathways in responders (Table S4, Sheet 5). In addition, pathways related to “tRNA processing” and to “RNA polymerase I and III transcription initiation” were upregulated. The pathways uniquely downregulated in responders (*n* = 184) were represented by, e.g., “actin dynamics for phagocytic cup formation”, “FCGR3A-mediated phagocytosis”, “Toll-like receptor activating cascades”, “platelet activation and degranulation”, and “detoxification of reactive oxygen species”.

In non-responders, 156 pathways in total (including the 22 above) were uniquely downregulated at follow-up (e.g processes of the “cell cycle” and “DNA replication”, “anaphase promoting complex mediated degradation of cell cycle proteins” and “interferon signaling”), but only one pathway (“protein–protein interactions at synapses”) was uniquely upregulated (Table S4, Sheet 5). 

Thirty-seven pathways were downregulated in both responders and non-responders, e.g., “antigen processing-cross-presentation”, “the complement cascade”, “scavenger receptors”, “antimicrobial peptides”, “interferon γ signaling” and “interferon α, β signaling” (Table S4; Sheet 5).

## 3. Discussion

Information is sparse on biomarkers for guiding treatment decision in patients with inflammatory bowel disease when contemplating treatment with VDZ. Here, we used peripheral whole blood in an attempt to identify candidate genes or candidate biological pathways for further evaluation. 

Our observation of there being no DEGs between UC and CD is in agreement with some reports [18], although it is in contrast with others [19]. However, in the gene-by-gene comparisons, the transcriptional dynamics were strikingly different between responders and non-responders following treatment. A large number of DEGs was observed in patients responding to VDZ compared with baseline. The eight most upregulated genes have been described in the contexts of cellular proliferation, differentiation and cellular apoptosis/survival, inhibition of NFKB activation, tissue development, and glucose metabolism [20,21,22,23,24,25]. We did not find any individual DEGs in the whole blood of non-responders at follow-up when compared with baseline. This is in agreement with observations in the intestinal mucosa of non-responders to VDZ [10,11], even though the literature is inconsistent on this point [12]. 

The investigation of the pre-treatment gene signatures did not reveal any DEGs between responders and non-responders which are suitable for use as predictive biomarkers. However, using colonic biopsies from patients naïve to biological treatment, Verstockt et al. [26] established a four-gene-based model which showed promising results in predicting endoscopic remission in response to VDZ. These four genes (*RGS13*, *DCHS2*, *MAATS1* and *PIWIL1*) were all expressed at low levels in blood and were excluded by the filtering of our data. Even though no individual genes were identified as suitable biomarkers, treatment outcomes may depend on several interacting genes and our results indicate the possible potential for pathway analysis in predicting response to VDZ. 

Although VDZ is considered gut-specific, the trafficking of leukocytes to the intestine as well as the relative and absolute abundance of peripheral blood cells seem unaffected by the drug [11,27,28]. These observations were largely confirmed in our study. However, in contrast to previous observations, we found a small but significant reduction in the relative abundance of B cells at follow-up with VDZ in responders. This was paralleled with a reduced expression of *MZB1*, which is related with B-cell activation and infiltration and immunoglobulin folding and secretion [29], as well as with other immunoglobulin-related genes. Indeed, VDZ binds to α_4_β_7_ on B cells [30], but little is known about the effect of this binding. Our results are consistent with previous reports, suggesting the potential importance of the B-cell population and signaling through α_4_β_7_ as well as of the B-cell receptor following VDZ [11,31,32,33]. However, the baseline enrichment of the sub-population of naïve B cells, noticed in the intestinal mucosa of patients responding with endoscopic remission to VDZ [26], was not observed in blood of our patients. 

Differences between responders and non-responders most likely depend on several interacting cell types and genes, each contributing with an individual small effect. This was illustrated by the identification of significant differences in pathway enrichment between groups, both at baseline and in response to VDZ. Zeissig et. al. [11] showed a dampening of innate immunity processes in intestinal biopsies of remitters to VDZ, which was confirmed by our findings in peripheral blood of responders when compared with baseline. In addition to the observed downregulation of pathways associated with “FC gamma receptor mediated phagocytosis” and “regulation of the actin dynamics for phagocytic cup formation”, pathways associated with “Toll-like receptors activation” (a crusial part of the innate immunity) were downregulated in responders. Toll-like receptors sense and recognize specific molecular patterns of microbial products, and their downstream signaling is also involved in regulating the adaptive immune cells implicated in the pathogenesis of IBD [34]. During inflammation, the production of reactive oxygen species by leukocytes and macrophages increases, whereas the production of antioxidant molecules decreases, creating a cellular imbalance [35,36]. In responders, pathways of “detoxification of reactive oxygen” species were downregulated, as were the pathways of “platelet degranulation and activation”, following VDZ treatment. Overall, this implies a dampening of innate inflammatory activity.

Even if expression of the α_4_β_7_ integrin is highest on memory CD4+ T cells, it is also expressed by many other cell populations [30]. The recruitment of cells of the innate immune system, such as monocytic progenitor dendritic cells, to the intestine rely on the α_4_β_7_-MadCAM-1 interaction [37,38]. These observations imply that the blockage of recruitment of innate immune cells may contribute to the effects of VDZ, in addition to its more well described effect on T cells. Furthermore, it was recently shown that α_4_β_7_^-^ T cells may enter the intestinal mucosa via inflammation-induced alternative mechanisms (e.g., via α_4_β_1_/VCAM-1 or αLβ_2_/ICAM-1) and differentiate to α_E_β_7_ effector cells, and that α_4_β_7_ blockade only partially reduces T cell trafficking to the gut. Therefore, it has been suggested that α_4_β_7_-directed therapy alone may leave additional compensatory homing mechanisms active [39,40], which merits further investigation. Blood-based transcriptomic profiling of treatment-naïve as well as treated IBD patients has shown an enrichment of pathways of “innate immunity”, and especially of myeloid-mediated immunity such as “neutrophil mediated immunity”, as well as the activation of “oxidative phosphorylation” when compared with healthy controls [18].

Following treatment with VDZ, “tRNA and rRNA processes” involved in the translational machinery, “mitochondrial translation”, and the process of “peroxisomal membrane protein import” were upregulated in responders compared with baseline values, but they were downregulated in non-responders. The upregulation of translational, mitochondrial translational and peroxisome-related pathways, as well as those associated with the transcription of RNA polymerase I and III following VDZ treatment, may indicate a restoration of the cellular milieu and functions compared with baseline in responders, but not in non-responders. The mitochondria regulate vital cellular functions besides energy production. Mitochondrial dysfunction, including increased oxidative stress and impaired ATP production, are characteristics of IBD [41]. Both rRNA synthesis and the ribosome production are energy-expensive processes, and in addition comprise critical and pleitropic elements involved in the control of cell growth and proliferation. The response to cellular stress is most commonly achieved by downregulating these processes, ultimately leading to aberrant protein synthesis and regulation of the cell cycle progression [42,43]. The peroxisome is, together with the mitochondria compartment, also responsible for the fatty acid beta-oxidation [44]. The observations here are consistent with our previous findings [45], where pathways of the mitochondrial respiratory chain and peroxisome were downregulated in the inflamed UC mucosa, whereas mitochondrial translation was upregulated in the noninflamed mucosa.

In our population, CRP was higher in responders than in non-responders, both before initiation of VDZ and at follow-up. However, the median CRP concentration did not decrease over time, either in responders or in non-responders, indicating that CRP was not associated with clinical response. Several studies have reported that a high baseline CRP may predict a better response to treatment with anti-TNFs, and in our data a similar observation was made. Furthermore, we consider it plausible that the absent decrease in CRP during treatment, in contrast to anti-TNF-α therapy, can be explained by the various modes of drug action. TNF-α directly upregulates IL6 production, and anti-TNF-α therapy accordingly results in a reduction in CRP, whereas similar decreases may not be seen for agents that do not directly block TNF-α, such as VDZ, which has been reviewed in [46,47]. In analogy with our observations, a reduction in CRP was not seen in the randomized trial of VDZ in UC [6].

The strength of our study is that it was performed on patients in a ‘real-world setting’ which were treated according to established clinical praxis in accordance with national and international guidelines, rather than a formal study protocol. There are some limitations, such as a small number of patients, which usually is the case in this kind of laborsome experimental set-up [10,11,12,26,48], which could affect the results. The estimation of appropriate group sizes and study power rely extensively on previous knowledge and are affected by biological variation, fold-change and the significance level selected [49]. Gene expression profiling in patients with different outcomes on biological drugs, including VDZ, has been performed previously, but mainly on isolated cell populations or intestinal biopsy samples [10,12,26,48,50]. In the responders, 201 genes were indeed regulated (33 with FC > 2). Even though our pathway analyses showed promising results, our data also indicated that the use of blood as matrix is not suitable for identifying single or a few candidate genes able to predict response to VDZ already at baseline. 

In conclusion, even if considered a gut-specific drug, our study shows that VDZ has considerable effects on the transcriptional profile in blood of patients responding to treatment, whereas no specific gene regulation was observed in non-responders. It also indicates that whole blood is not optimal for identifying predictive pre-treatment biomarkers based on individual genes in patients with inflammatory bowel disease and in need for this integrin inhibitor. However, our results suggest the potential of pathway analysis in predicting responses to treatment, illustrated by the identification of significant effects on pathways associated with innate immune response, as well as on ribosomal and mitochondrial translation, and the peroxisome. These effects were closely associated with the response to treatment. The use of newer technologies, such as single-cell RNA-sequencing, may possible shed more light on the contribution of different cell types to the efficacy.

## 4. Materials and Methods

### 4.1. Study Population

Twenty-four patients > 15 years of age (9 UC, 15 CD) from the outpatient clinics at the Division of Gastroenterology, Karolinska University Hospital in Stockholm, Sweden, who previously had failed to respond to at least one anti-TNF-α drug, were included. 

Demographic- and disease-related data, including age, gender, disease duration, concomitant medication and routine laboratory test results, were retrieved from the patients’ medical records. At inclusion, 15 patients were on glucocorticosteroids. Nine patients were treated with mesalazine and nine with thiopurines, and the doses of these drugs were unchanged over the study period. All patients were naïve to VDZ. No patient had received ustekinumab. Sample group sizes were based on the recommendations in the work of Schurch et al., 2017, and Bi et al., 2016 [51,52].

Patients were sampled at baseline before starting VDZ (T0) and at follow-up at week 10–12 (T1), when clinical efficacy was assessed. At this point, all patient had received three infusions (300 mg each) of VDZ at week 0, 2 and 6, respectively. A sHBI (abdominal palpation excluded) ≤ 4 or a SCCAI ≤ 2 was used to define clinical remission. Clinical response was defined as a decrease in either sHBI ≥ 3 or SCCAI ≥ 3 from baseline [53,54,55] or as a ‘physician global assessment’ by one experienced gastroenterologist without access to endoscopy or laboratory results (taking presence of diarrhea, stool frequency, abdominal pain, fatigue, presence of fever and weight into account). Here, grade 0 was remission, grade 1 mild, grade 2 moderate, and grade 4 severe disease activity.

### 4.2. Sample Collection

Blood samples were collected at T0 and T1. Plasma was isolated from whole blood collected in standard K_2_EDTA tubes (Becton, Dickinson and Company, Franklin Lakes, NJ, USA) and stored at −70 °C until analysis. For the isolation of RNA, blood was collected and stabilized in Tempus Blood RNA tubes (ThermoFisher Scientific, Waltham, MA, USA) [56].

### 4.3. Plasma Concentration of Vedolizumab

The plasma concentration of VDZ was determined at T1 (week 10–12) with an in-house enzyme-linked immunosorbent assay method used in clinical routines at the Department of Clinical Immunology and Transfusion medicine, Karolinska University Laboratory, Stockholm, Sweden. The imprecision at VDZ 1.4 µg/mL and 7.3 µg/mL was 30% and 20%, respectively.

### 4.4. RNA Sequencing

RNA sequencing was performed at the National Genomics Infrastructure (NGI), SciLifeLab, Stockholm, Sweden. Libraries were prepared with TruSeq Stranded Total RNA with ribosomal depletion using Ribo-Zero Globin (Illumina Inc, San Diego, CA, USA) following the manufacturer’s protocols, after which they were sequenced on NovaSeq6000 (Illumina) with a read length of 2 × 51.

### 4.5. Statistical Analyses

#### 4.5.1. Basic Statistics

Basic statistics were calculated using Statistica v.13.3 (Statsoft Inc, Tulsa, OK, USA) and presented as median (IQR) values. The paired sample Wilcoxon test was used when comparing dependent samples and the Kruskal–Wallis one-way analysis of variance by ranks was used when comparing independent groups. For categorical variables, Fisher’s exact test was used. In all comparisons, two-sided tests were used and considered statistically significant if *p* ≤ 0.05.

#### 4.5.2. Differentially Expressed Genes

Analysis of DEGs was performed as previously described [56,57], using packages from the Bioconductor project; Rsubread v.2.0.1, edgeR v.3.28.1, limma v.3.42.2, in RStudio v.1.3.1073 [58] and R v.3.6.0. Genes without annotation were excluded, and only genes with expression > 2 counts per million (cpm) in at least nine samples (corresponding to the smallest study group) were retained. Sample differences and similarities were visualized using multidimensional scaling based on the top 500 genes with the largest variance between all samples and the Glimma package v.1.14.0 [59]. The duplicateCorrelation function in limma [60] was applied, before linear modeling with a Bayesian moderated *t* test, to assess significant differences from zero for each group contrast. Thus, no fold-change (FC) cut-off was used when establishing significant results. The *p* values were corrected for false discovery rate (FDR) according to Benjamini and Hochberg [61], and genes with corrected *p* values < 0.05 were considered to be significantly DEGs.

#### 4.5.3. Pathway Analyses

Significant DEGs were investigated for their biological context by over-representation analysis using the R package ReactomePA v.1.3.0 [62]. DEGs were further explored for biological context by means of quantitative text analysis of functional protein information (we manually reviewed UniProtKB/Swiss-Prot database records) associated with the corresponding genes, as previously described by our group [45]. Further, ranked lists of all analyzed genes (in descending order) were computed for each pairwise group comparison by multiplying the direction (sign) of the fold change with the absolute value of the logarithm of the corresponding *p* value, thus generating lists with upregulated genes at the top and downregulated genes at the bottom. The ranked lists were used for gene set enrichment analysis (GSEA) of reactome [63] biological pathways using the GSEA software v.4.1.0 [64], with 10,000 gene set permutations and gene sets versions as of 1 July 2020. An FDR of 0.075 and an absolute normalized enrichment score > 2 were used as the cutoff point when results were visualized using EnrichmentMap v.3.3.1 [65] in Cytoscape v.3.8.0 [66], applying a Jaccard similarity cut off of 0.5. AutoAnnotate v.1.3.3 and clusterMaker v.1.3.1 with the Markov clustering algorithm [67] was used to identify and annotate clusters.

#### 4.5.4. Deconvolution

The proportion of the various peripheral white blood cell types was predicted using RNA sequencing data, expressed as log2-transformed transcripts per million, and immunoStates deconvolution analysis in MetaIntegrator v.2.1.5 (kindly provided by Aditya Rao, Stanford University, Stanford, CA, USA) [68]. The resulting relative abundance of cell populations was investigated for differences between groups.

## Figures and Tables

**Table 1:** Patient characteristics at baseline (T0) and following vedolizumab (T1), *n* = 24 (9 UC, 15 CD).

		Responders (*n* = 13)	*p*-Value T0-T1	Non-Responders (*n* = 11)	*p*-Value T0–T1	*p*-Value at T0 or T1
Disease UC/CD		6/7		3/8		0.42
Gender (female/male)		4/9		3/8		1.00
Age (years)		30.2 (16.6)		37.6 (30.2)		0.28
Disease duration (years)		10.2 (13.5)		16.3 (15.2)		0.26
Days since last anti-TNF-α drug ^†^		77 (1593)		99 (763)		0.98
Duration last anti-TNF-α drug (days) ^†^		202 (356)		267 (903)		0.10
Disease activity UC	T0	11 (3)		10 (5)		0.52
	T1	6 (6)	0.02	8 (9)	1.00	0.52
	6 months ^‡^	3 (4)	0.09	8 (15)	0.79	1.00
Disease activity CD	T0	10 (5)		7 (5)		0.14
	T1	4 (4)	0.03	8 (6)	0.55	0.28
	6 months ^§^	6 (3)	0.07	6 (9)	0.11	0.91
Clinical remission	T0	1		2		0.57
	T1	4		2		0.65
PGA	T0	2 (0)		2 (0)		0.69
	T1	1 (1)	0.04	2 (1)	0.11	1.00
f-Calprotectin (mg/kg feces)	T0 ^¶^	1390 (2112)		549.5 (877)		0.11
	T1 ^††^	191 (1417)	0.06	266 (269)	0.58	0.96
s-CRP (mg/L)	T0	9.0 (21.0)		3.0 (5.0)		0.02
	T1	13.0 (14.0)	0.23	3.0 (1.0)	0.83	0.01
b-Leukocyte count (×10^9^/L)	T0	9.2 (4.1)		9.2 (4.0)		0.86
	T1 ^‡‡^	8.35 (3.0)	0.16	7.8 (3.4)	0.32	0.98
b-Hb (g/L)	T0	130 (17)		133 (22)		0.57
	T1 ^§§^	125 (25)	0.39	140 (25)	0.79	0.35
s-Alb (g/L)	T0	35 (6)		37 (6)		0.57
	T1	35 (1)	0.48	36 (4)	0.62	0.46
Dose VDZ (mg/kg body weight)						
		4.1 (0.47)		3.9 (1.2)		0.65
p-VDZ at follow-up (T1) (µg/mL)		10.5 (9.9)		16.2 (8.1)		0.19

Data presented as median (IQR). UC: ulcerative colitis; CD: Crohn’s disease; s: serum; p: plasma; f: fecal. Disease activity: Simple Clinical Colitis Activity Index (SCCAI) for UC and Simplified Harvey Bradshaw index for CD (sHBI). Clinical remission was defined as sHBI ≤ 4 or SSCAI ≤ 2. PGA: Physician global assessment; CRP: C-reactive protein; Hb: hemoglobin; Alb: albumin; VDZ: vedolizumab. T0: baseline before vedolizumab infusion. T1: follow-up after 10–12 weeks with vedolizumab. ^†^ data missing in one responder. ^‡^ comparison with T1, data available in six responders and three non-responders still on VDZ. ^§^ comparison with T1, data available in four responders and five non-responders still on VDZ. ^¶^ data missing in four responders and one non-responder. ^††^ data missing in five responders and in one non-responder. ^‡‡, §§^ data missing in one responder.

**Table 2:** The number of enriched biological pathways, with a false discovery rate adjusted *p* value < 0.075, in the gene set enrichment analysis based on all pairwise statistical comparisons.

Pathway Enrichment	Reactome	
	Up	Down
T0 Responders vs. Non-responders	279	46
T1 Responders vs. Non-responders	33	7
Responders T1 vs. T0	51	221
Non-responders T1 vs. T0	1	193

T0; baseline before initiating vedolizumab. T1; follow up after 10–12 weeks of vedolizumab.

**Table 3:** The most enriched reactome pathways with an absolute NES > 2 in the comparison between transcriptional profiles in responders (*n* = 9) and non-responders (*n* = 11) at baseline.

**Pathways Upregulated in Responders**	**Size**	**NES**	**FDR *p*-Value**
Amino acid transport across the plasma membrane	19	2.30	2.44 × 10^−4^
Regulation of actin dynamics for phagocytic cup formation	61	2.27	8.12 × 10^−5^
FCGR3A-mediated phagocytosis	58	2.27	4.87 × 10^−5^
Glycosaminoglucan metabolism	70	2.18	3.65 × 10^−4^
EPH-Ephrin signaling	66	2.13	8.33 × 10^−4^
**Pathways Downregulated in Responders**			
Mitochondrial translation initiation	82	−2.41	<1.00 × 10^−5^
Mitochondrial translation termination	82	−2.40	<1.00 × 10^−5^
Mitochondrial translation	88	−2.40	<1.00 × 10^−5^
tRNA processing	93	−2.26	3.10 × 10^−5^
rRNA processing in the nucleus and cytosol	171	2.05	<1.00 × 10^−5^

FDR: false discovery rate; NES: normalized enrichment score.

**Table 4:** The most enriched reactome pathways with an absolute NES ≥ 2 in the comparison between transcriptional profiles in responders (*n* = 9) and non-responders (*n* = 11) at follow-up with vedolizumab.

**Pathways Upregulated in Responders**	**Size**	**NES**	**FDR *p*-Value**
L13-mediated translational silencing of ceruloplasmin expression	107	2.28	2.67 × 10^−4^
GTP hydrolysis and joining of the 60S ribosomal subunit	108	2.26	2.26 × 10^−4^
Eukaryotic translation elongation	87	2.25	2.42 × 10^−4^
Peptide chain elongation	85	2.24	2.04 × 10^−4^
Viral mRNA translation	85	2.21	2.35 × 10^−4^
**Pathways Downregulated in Responders**			
Interferon alpha beta signaling	49	−2.11	1.03 × 10^−2^
Antigen processing cross-presentation	94	−1.98	5.31 × 10^−2^
Interferon gamma signaling	75	−1.97	4.15 × 10^−2^
ADP signaling through P2Y purinoceptor	14	−1.90	7.14 × 10^−2^
ER-phagosome pathway	80	−1.89	7.01 × 10^−2^

FDR; false discovery rate, NES; normalized enrichment score.

## Data Availability

Research materials supporting this publication are not publicly available but may be accessed after reasonable motivation by contacting the corresponding author. The data are not publicly available due to regulation regarding information that potentially could identify and compromise the privacy of research participants.

## Supplementary Materials

The following supporting information can be downloaded at: https://www.mdpi.com/article/10.3390/ijms24065820/s1.

## Abbreviations

IBD	inflammatory bowel disease
UC	ulcerative colitis
CD	Crohn’s disease
VDZ	vedolizumab
sHBI	simplified Harvey Bradshaw index
SCCAI	Simple Clinical Colitis Activity Index
PGA	physician global assessment
DEGs	differentially expressed genes
FC	fold-change
FDR	false discovery rate
ORA	over-representation analysis
GSEA	gene set enrichment analysis
NES	normalized enrichment score
CRP	C-reactive protein
Hb	haemoglobin
Alb	albumin
