# Supplementary material for: Differences in Whole-Blood Transcriptional Profiles in Inflammatory Bowel Disease Patients Responding to Vedolizumab Compared with Non-Responders
Source: Int J Mol Sci. 2023 Mar 18;24(6):5820. doi: 10.3390/ijms24065820 (PMC10052064; doi:10.3390/ijms24065820)
Supplement: Supplementary file 1 [file ijms-24-05820-s001.zip › Supplementary Table S1.pdf]

Supplementary Table S1. Description of patients included in RNA-sequencing with paired RNA samples representing baseline (T0) and follow-up (T1), n = 20 (7 UC, 13 CD).

		**Responders** (n = 9)	***P*-value T0-T1**	**Non-responders** (n = 11)	***P*-value T0-T1**	***P*-value at T0 or T1**
Disease UC/CD		4 / 5		3 / 8		0.64
Gender (female/male)		3 / 6		3 / 8		1.00
Age (years)		31.6 (16.6)		37.6 (30.2)		0.26
Disease duration (years)		9.6 (11.5)		16.3 (15.2)		0.26
Days since last anti-TNF-α drug†		204 (1578)		99 (763)		0.60
Duration last anti-TNF-α drug (days) †		243 (324)		267 (903)		0.31
Smoker (yes/no)		9 / 0		9 / 2		0.48
5ASA (T0/T1)		5 / 5		2 / 2		1.00
Corticosteroids (T0/T1)		7 / 4		9 / 5		1.00
Thiopurines (T0/T1)		2 / 1		1 / 1		1.00
Disease activity UC	T0	10 (8)		10 (5)		0.86
	T1	5 (11)	0.07	8 (9)	1.00	0.63
	6 months‡	4 (5)	0.42	8 (15)	0.79	0.86
Disease activity CD	T0	9 (5)		7 (5)		0.22
	T1	3 (3)	**0.04**	8 (6)	0.55	0.35
	6 months§	6 (2)	0.11	6 (9)	0.11	1.00
Clinical remission (n)	T0	1		2		1.00
	T1	4		2		0.34
Physician global assessment	T0	2 (0)		2 (0)		0.82
	T1	1 (1)	**0.02**	2 (1)	0.11	0.41
f-Calprotectin (mg/kg feces)	T0¶	979 (2267)		549.5 (877)		0.31
	T1††	841 (2479)	0.07	266 (269)	0.58	1.00
s-CRP (mg/L)	T0	9.0 (30)		3.0 (5.0)		0.08
	T1	13.0 (16.0)	0.40	3.0 (1.0)	0.83	0.07
b-Leukocyte count ($x10^9/L$)	T0	9.2 (5.2)		9.2 (4.0)		0.88
	T1‡‡	7.5 (2.6)	0.21	7.8 (3.4)	0.32	0.60
b-Hb (g/L)	T0	137 (25)		133 (22)		0.88
	T1§§	125 (42)	0.33	140 (25)	0.79	0.72

s-Alb (g/L)	T0	37 (4)		37 (6)		0.77
	T1	35 (1)	0.67	36 (4)	0.62	0.66
Dose VDZ (mg/kg body weight)		3.8 (0.43)		3.9 (1.2)		0.46
p-VDZ (µg/mL)		10.5 (9.9)		16.2 (8.1)		0.23

Data presented as median (IQR). UC; ulcerative colitis. CD; Crohn's disease. s; serum. p; plasma. f; fecal. Disease activity; Simple Clinical Colitis Activity Index (SCCAI) and Simplified Harvey Bradshaw index (sHBI). Clinical remission sHBI ≤ 4 or SSCAI ≤ 2. CRP; C-reactive protein. Hb; hemoglobin. Alb; albumin. VDZ; vedolizumab. T0; baseline before vedolizumab infusion. T1; follow-up after 10 weeks with vedolizumab. [†]Data missing in one responder, [‡]Comparison with T1, data available in three responders and three non-responders still on VDZ, [§]comparison with T1, data available in three responders and five non-responders still on VDZ, [¶]data missing in three responders and one non-responder, [††]data missing in five responders and in one non-responder, [‡‡,§§]data missing in one responder.
