# Supplementary material for: Differences in Whole-Blood Transcriptional Profiles in Inflammatory Bowel Disease Patients Responding to Vedolizumab Compared with Non-Responders
Source: Int J Mol Sci. 2023 Mar 18;24(6):5820. doi: 10.3390/ijms24065820 (PMC10052064; doi:10.3390/ijms24065820)
Supplement: Supplementary file 1 [file ijms-24-05820-s001.zip › Supplementary Table S2.pdf]

Supplementary Table S2: Mean relative cellular abundance as estimated by deconvolution analysis. Differences in relative abundances between groups were assessed using Kruskal-Wallis one-way analysis of variance by ranks. When more than 50% of samples lacked data, the cell-type was excluded from analysis.

	CD4+ alpha beta T cell	Memory B cell	Neutrophil	Natural killer cell	Monocyte	B cell	T cell	Granulocyte
**Responders**								
T0	0.157	0.041	0.338	0.135	0.236	0.047	0.159	0.338
T1	0.179	0.029	0.295	0.160	0.217	0.034	0.185	0.295
P-value T1 *vs*. T0	0.546	0.182	0.505	0.182	0.182	**0.046**	0.182	0.505
**Nonresponders**								
T0	0.159	0.033	0.319	0.158	0.233	0.041	0.180	0.319
T1	0.160	0.028	0.330	0.166	0.234	0.034	0.169	0.331
P-value T1 *vs*. T0	0.546	0.228	1.000	1.000	0.546	0.227	1.000	1.000
P-value 4-group comparison	0.753	0.627	0.922	0.744	0.962	0.616	0.578	0.915

T0; baseline before initiating vedolizumab. T1; follow up after 10-12 weeks of vedolizumab treatment

The following cells were estimated to very low relative abundance:
CD8+ alpha beta cells were detected in 4/9 responders and in 4/11 non-responders.
Naïve B-cells were detected in 1/9 responders and in 7/11 non-responders.
Plasma cells were detected in 6/9 responders and in 3/11 non-responders
